# Study on Tullbergiidae of Tibet, China I. *Metaphorura*, *Mesaphorura* and *Prabhergia* (Hexapoda, Collembola)

**DOI:** 10.3897/zookeys.686.11468

**Published:** 2017-07-24

**Authors:** Yun Bu, Yan Gao

**Affiliations:** 1 Natural History Research Center, Shanghai Natural History Museum, Shanghai Science & Technology Museum, Shanghai, 200041, China; 2 Shanghai Hengjie Chemical Co. Ltd., Shanghai, 201599, China

**Keywords:** chaetotaxy, Motuo, postantennal organ, pseudocelli

## Abstract

The Tullbergiidae of Tibet is studied for the first time and the genus *Metaphorura* Bagnall, 1936 is firstly recorded in China. *Metaphorura motuoensis* **sp. n.** from southeastern Tibet is described and illustrated. It is characterized by the presence of 1+1 pseudocelli on thoracic segment I, few vesicles (14 -16) on PAO, pseudocellar formula as 11/111/11111, all pseudocelli of type II, setae p4 on abdominal segment V as microsetae, weakly differentiated sensory seta p3 on abdominal segment V, absence of median process on Abd VI. In addition, *Mesaphorura yosii* (Rusek, 1967), *Mesaphorura hylophila* Rusek, 1982, and *Prabhergia imadatei* Tamura & Zhao, 1996 are recorded in Tibet for the first time. The type specimens of *P. imadatei* are re-examined and errors in the original description of chaetotaxy are corrected.

## Introduction

The knowledge of the taxonomy of Tullbergiidae from China is quite insufficient and only five species have been recorded to date (Rusek 1967; Tamura and Zhao 1996; Gao 2007; Bu et al. 2013; Bu and Gao 2015).There are no records of Tullbergiidae from Tibet. During an investigation of soil arthropods in southeast Tibet in November 2015, nearly 200 specimens belonging to the family Tullbergiidae were obtained. They were identified as four species including one new species of the genus *Metaphorura* Bagnall, 1936. The new species is illustrated and described in the present paper.

## Materials and methods

Specimens were collected by Berlese-Tullgren funnels and preserved in 80% ethanol. The material was mounted on slides in Hoyer’s solution and dried in an oven at 60 °C for identification. Observations were done with a phase contrast microscope. Photos were taken by a digital camera installed on the microscope. The type specimens are deposited in Shanghai Natural History Museum (SNHM), Shanghai, China. In the description we use the nomenclature for morphological features following Dunger and Schlitt (2011). Pseudocellar types after Weiner and Najt (1991). Antennal chaetae notation is made following the notation of Rusek (1971). Formula of tibiotarsal chaetotaxy follows Fjellberg (1991).

Abbreviations used in the descriptions:

**Th.** thoracic segment,

**Abd.** abdominal segment,

**Ant.** antennal segment,

**Asp.** anal spine,

**s** sensillum,

**PAO** postantennal organ,

**a** anterior setae,

**m** medial setae,

**p** posterior setae,

**pl** ,

**pso** pseudocelli.

## Results

### Taxonomy

#### Genus Metaphorura

Taxon classificationAnimaliaCollembolaTullbergiidae

Bagnall, 1936

##### Diagnosis.

Habitus more or less slender. Granulation of the integument moderately fine, with stronger granulation only on head and areas of Abd VI. Usually no crescentic ridges. A median process usually present ventrally between Asp. Antennal segment III with two large sensory clubs, bent towards one another, two small sensory rods and three protecting papillae. Postantennal organ with 14–28 vesicles in two rows. Pseudocelli clearly delimited, with two rows of parallel stripes in the centre (type II). Asp strong, distinctly longer than the claw of leg III.

##### Distribution.

The genus *Metaphorura* includes nine species found in Holarctic regions (Dunger and Schlitt 2011; Bellinger et al. 1996–2017). It is newly recorded from China in this paper.

#### Metaphorura motuoensis sp. n.

Taxon classificationAnimaliaCollembolaTullbergiidae

http://zoobank.org/A71C4114-150F-4A47-9F13-85C7D712B1B8

Figs 1–16 , 17–19 , 20–24 , Table 1

##### Material examined.

Holotype, female (slide no. XZ-C2015014) (SNHM), China, Tibet, Motuo county, Dexing town, extracted from soil samples in broad-leaved forest, Alt. 1100 m, 29°40'N, 95°26'E, 3-XI-2015, coll. Y. Bu & G. Yang. Paratypes, 8 females (slides nos. XZ-C2015008–XZ-C2015013, XZ-C2015015, XZ-C2015037) (SNHM), 10 males (slides nos. XZ-C2015016 –XZ-C2015025), same data as holotype. Other material: 1 male (slide no. XZ-C2015095) (SNHM), China, Tibet, Linzhi, Sejila Mt., extracted from soil samples under bushes, Alt. 3500 m, 29°67'N, 95°70'E, 1-XI-2015, coll. Y. Bu & G. Yang; 17 juveniles (slides nos. XZ-C2015026, XZ-C2015027, XZ-C2015106–XZ-C2015112) (SNHM), 45 adults in alcohol, same data as holotype.

**Table 1.** Dorsal Chaetotaxy of *Metaphorura motuoensis* sp. n.

Row	Thorax			Abdomen				
	I	II	III	I	II	III	IV	V
a	-	10	10	10	10	10	8^3^	10^5^
m	8	8	8	2^1^	4^2^	4^2^	4^2^	-
p	-	8	8	10	10	10	8^4^	8^6^
pl	2	3	3	2	3	3	6	1

^1^ seta m4 present; ^2^ seta m3 and m4 present; ^3^ seta a5 absent; ^4^ seta p4 absent; ^5^ seta a2 and p4 as microseta; ^6^ sensory seta p3 slightly differentiated, seta p4 as microseta.

##### Diagnosis.

*Metaphorura motuoensis* sp. n. is characterized by the presence of pseudocelli on thoracic segment I, few simple vesicles (14–16) on PAO, pseudocellar formula as 11/111/11111, all pseudocelli of type II, p4 on abdominal segment V as microsetae, less differentiated sensory setae p3 on abdominal segment V, absence of median process on Abd VI. Bisexual.

##### Description.

**Figures 1–16.**
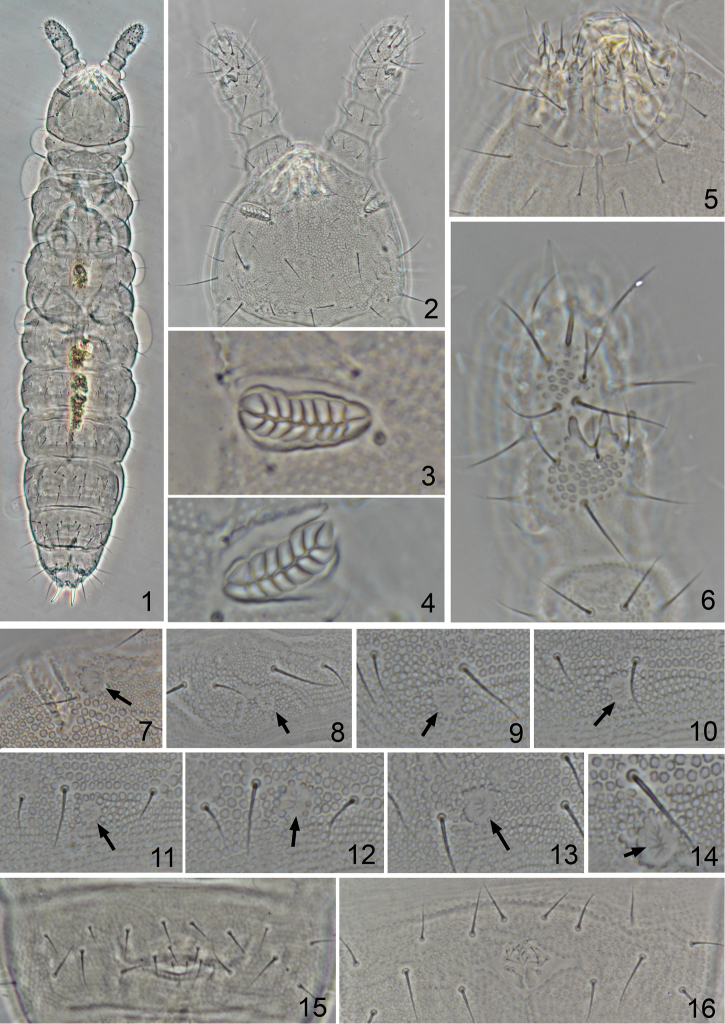
*Metaphorura motuoensis* sp. n. (Holotype) **1** Habitus, dorsal view **2** head, dorsal view **3** Left postantennal organ **4** Right postantennal organ (paratype XZ-C2015016) **5** Labium **6** Antenna, segments II–IV, dorsal view **7** Pseudocellus on anterior of head **8** Pseudocellus on Th. I **9** Pseudocellus on Th. II **10** Pseudocellus on Th. III **11** Pseudocellus on Abd. I **12** Pseudocellus on Abd. II **13** Pseudocellus on Abd. IV **14** Pseudocellus on Abd. V **15** Female genital plate **16** Male genital plate (paratype XZ-C2015021). Arrows in figs 7–14 indicate the pseudocelli.

Adult *body* 0.86 mm long in average (0.72–0.95 mm, n = 19), holotype 0.93 mm (Fig. 1). Both female and male were present. Setae well differentiated into micro- and macrosetae. Granulation of integument moderately fine (2.5–3.0 μm), with stronger granulation only on head and Abd. VI (4–5 μm). (Figs 1, 2, 23). Pseudocellar formula: 11/111/11111. All pseudocelli composed by two rows of parallel stripes in the center (type II, Figs 7–14), 7–8 μm in diameter, on Th. I between seta m2 and m3, close to hind margin; on Th. II and III between setae p3/p4, close to p3; on Abd. I–III posterior to seta p3; on Abd. IV parallel to seta p3; on Abd. V on the border of Abd. VI.

*Head seta* a0 present (18–20 μm), c1 absent, oc2 as macroseta (23–25 μm), sd5 as mesoseta (18–21 μm) (Fig. 2). Postantennal organ 23–26 μm long, 7–8 μm wide, composed of 14–16 elliptical vesicles arranged in two rows (Figs 2, 3), situated in a deep furrow (Fig. 7). Labrum with 4/4/2 setae. Labium with five papillae, six apical guard setae, six proximal setae, four basomedian setae, and five basolateral setae (Fig. 5). Ventral head with 3+3 axial setae.

**Figures 17–19.**
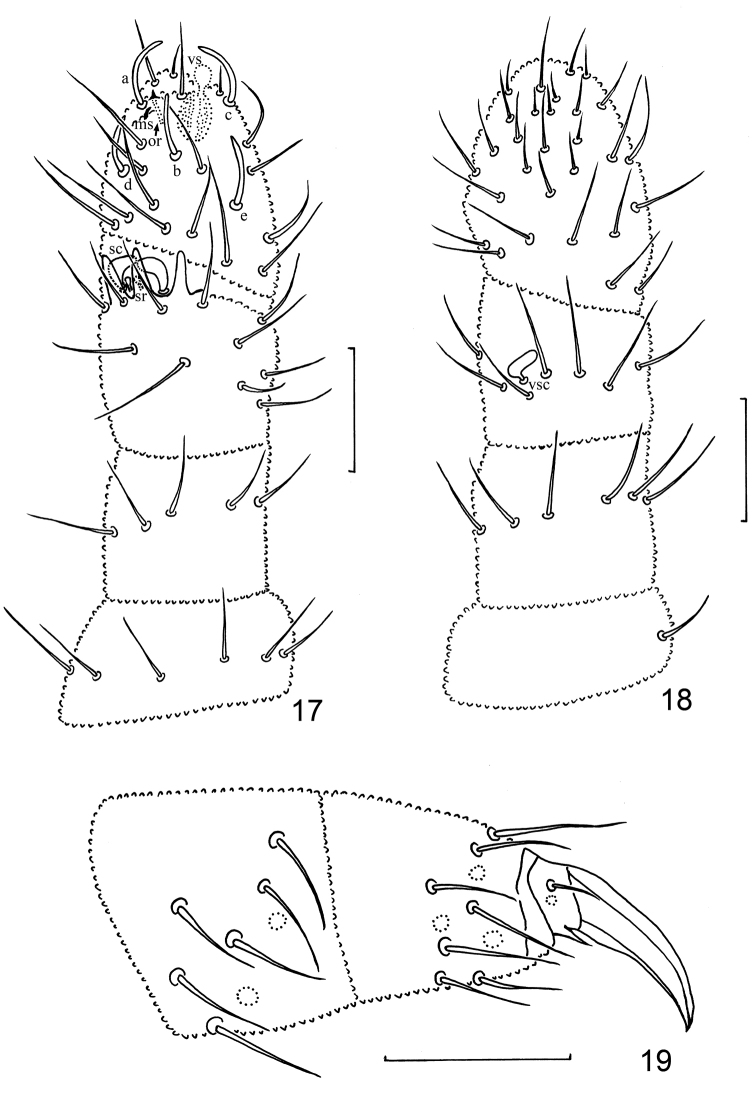
*Metaphorura motuoensis* sp. n. (Holotype) **17** Antenna, dorsal view, a, b, c, d, e–large sensilla, ms–microsensillum, or–subapical organite, vs– apical vesicles, sc–sensory clubs, sr–sensory rods **18** Antenna, ventral view, vsc–ventral sensory club **19** Leg III. Scale bars 20 μm.

*Antenna* (100–115 μm) shorter than head (130–135 μm). Ant. I and II with seven and eleven setae respectively (Figs 17, 18). Ant. segment IV with five slightly thickened sensilla a–e, sensilla a, c, e long and slightly curved toward inside, b and d slightly short (Fig. 17). Small microsensillum, subapical organite and one large apical vesicle present (Fig. 17). Antennal organ III with two small sensory rods between two thick sensory clubs bent toward each other, concealed behind three papillae and four guard setae (Figs 6, 17, 18).

*Legs* without clavate tenant hairs (Fig. 19). Subcoxa, coxa, trochanter, femur, and tibiotarsus with 0/3/3; 3/7/7; 5/5/4; 8/8/8; 10/10/10 setae on leg I, II and III, respectively (Fig. 19), tibiotarsi each with 6+4 setae (A1 to A6, B4 to B7, and M absent). Anal lobes with setae 12’ and l3’ (Fig. 24). Claw 20–23 μm long, untoothed, with short empodial appendage (3–4 μm).

**Figures 20–24.**
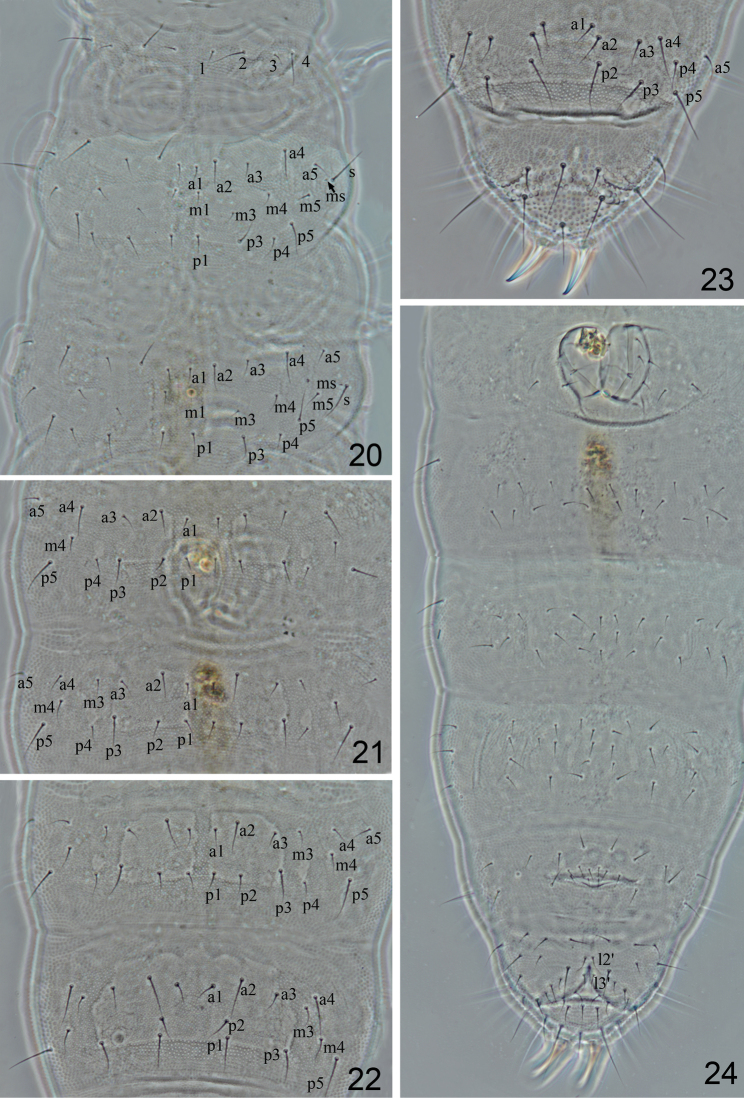
*Metaphorura motuoensis* sp. n. (Holotype) **20** Th. I–III, dorsal view, s–sensillum, ms–microsensillum **21** Abd. I–II, dorsal view **22** Abd. III–IV, dorsal view **23** Abd. V–VI, dorsal view **24** Abdomen, ventral view.

*Adult chaetotaxy* given in Figs 21–24 and Table 1. Setae on Th. I length as 10–13 μm for m1 and m3, 20–26 μm for m2 and m4 (Fig. 20). Microsensilla present on Th. II-III, and lateral sensory setae s 23–26 μm long (Fig. 20). Thorax with 0, 2, 2 ventral setae. Abd. I–III each with 2+2 axial setae dorsally, setae m4 present on Abd. I, setae m3 and m4 present on Abd. II–III. Abd. IV without seta px, setae m3 and m4 present. Abd. V with sensory seta p3 (24 μm) slightly differentiated; seta a2 as mesoseta (20–22 μm) and p4 as microsetae (Fig. 23). Crescentic ridges on Abd. VI present. Abd. VI with distinct dorsal secondary granulations, without median process between the anal spines (Fig. 23). Anal spines 28–32 μm long (Fig. 23).

Number of *ventral setae* on Abd. II, III and IV variable, with 18–20, 19–23, and 22–26 setae respectively (Fig. 24). Ventral tube with 4+4 apical setae and 2+2 basal setae (Fig. 24). Female genital plate with 3 pairs of pregenital setae, 2–3 pairs of circumgenital and one pair of eugenital setae (Fig. 15). Male genital plate with 3 pairs of pregenital setae, 6–9 pairs circumgenital setae and 1+1 eugenital setae (Fig. 16).

##### Etymology.

The species is named after the Motuo county where the type specimens were collected.

##### Distribution.

Known only from the type locality.

##### Remarks.

There are two genera of Tullbergiidae with two anal spines, three protecting papillae, three thick sensory clubs on antennal segment III, and an elongate PAO and body longer than 0.7 mm: *Delamarephorura* and *Metaphorura*. The new species better fits *Delamarephorura* in the presence of crescent ridges on Abd. VI, which are absent in *Metaphorura*, and the presence of a “median process” on Abd. VI which is present in *Metaphorura* (present or absent in *Delamarephorura*). However, according to Janion et al (2013): “the discrimination between *Delamarephorura* and *Metaphorura* needs to be re-evaluated” for uncertainties about the state of some diagnostic characters. The new species is therefore provisionally assigned to the genus *Metaphorura*, awaiting the validation of *Delamarephorura*. Biogeographically, Tibet is part of the Holarctic region where the genus *Metaphorura* is distributed, while *Delamarephorura* is only known in Africa and in Vietnam.

*Metaphorura motuoensis* sp. n. has few simple vesicles (14–16) on PAO and lacks the median process on Abd. VI which clearly separate it from other congeners. It is similar to *M. affinis* (Börner, 1902) in the presence of simple vesicles on PAO and the pseudocellar formula on the body, but differs in the presence of a crescentic ridge on Abd. VI, fewer vesicles on PAO (20–25 vesicles in *M. affinis*), two pairs of m-setae m3 and m4 on Abd IV (three pairs of m-setae in *M. affinis*: m2, m3, m4), and absence of median process on Abd. VI (presence of a pointed projection in *M. affinis*) and less differentiated sensory seta p3 on abdominal segment V (flame-like p3 in *M. affinis*). It is even more similar to *Delamarephorura capensis* Janion, Deharveng & Weiner, 2013, from which it differs by seta p1 on head much longer than p2 (versus of same size in *D. capensis*).

#### Mesaphorura yosii

Taxon classificationAnimaliaCollembolaTullbergiidae

(Rusek, 1967)

##### Material examined.

2 female, 1 juvenile (slides nos. XZ-C2015064, XZ-C2015065) (SNHM), China, Tibet, Linzhi, Sejila Mt., extracted from soil samples under bushes, Alt. 3500 m, 29°67'N, 95°70'E, 1-XI-2015, coll. Y. Bu & G. Yang; 7 females (slides nos. XZ-C2015007, XZ-C2015028, XZ-C2015029, XZ-C2015038, XZ-C2015073, XZ-C2015105, XZ-C2015134), 4 juvenile (slides no. XZ-C2015030, XZ-C2015073, XZ-C2015113) (SNHM), China, Tibet, Motuo county, Dexing town, extracted from soil samples in broad-leaved forest, Alt. 1100 m, 29°40'N, 95°26'E, 3-XI-2015, coll. Y. Bu & G. Yang. 32 females (slides nos. XZ-C2015039–XZ-C2015049, XZ-C2015075–XZ-C2015077, XZ-C2015142, XZ-C2015143), 3 juveniles (slides no. XZ-C2015050, XZ-C2015076) (SNHM) and 20 adults in alcohol, China, Tibet, Motuo county, Beibeng town, extracted from soil samples in broad-leaved forest, Alt. 1500 m, 29°30'N, 95°38'E, 5-XI-2015, coll. Y. Bu & G. Yang. 18 females (slides nos. XZ-C2015051, XZ-C2015052, XZ-C2015054, XZ-C2015085, XZ-C2015168–XZ-C2015172), 1 juvenile (No. XZ-C2015053) (SNHM), China, Tibet, Linzhi City, Bomi county, Songzong town, extracted from soil samples in broad-leaved forest, Alt. 3000 m, 29°76'N, 95°96'E, 7-XI-2015, coll. Y. Bu & G. Yang. 32 females (slides nos. XZ-C2015178–XZ-C2015184) (SNHM), China, Tibet, Linzhi City, Lulang forest farm, extracted from soil samples in coniferous forest, Alt. 2459 m, 29°98'N, 95°33'E, 9-XI-2015, coll. Y. Bu & G. Yang.

##### Distribution.

Cosmopolitan. In China the species was recorded in Fujian, Guangdong, Hunan, Shandong, Shanghai, Yunnan, Zhejiang, and for the first time in Tibet in this paper.

#### Mesaphorura hylophila

Taxon classificationAnimaliaCollembolaTullbergiidae

Rusek, 1982

##### Material examined.

11 females (slides nos. XZ-C2015083, XZ-C2015153, XZ-C2015154, XZ-C2015171, XZ-C2015172), 1 juvenile (No. XZ-C2015053) (SNHM), China, Tibet, Linzhi City, Bomi county, Songzong town, extracted from soil samples on broad-leaved forest, Alt. 3000 m, 29°76'N, 95°96'E, 7-XI-2015, coll. Y. Bu & G. Yang.

##### Distribution.

Widely distributed in Palaearctic region. In China the species was recorded in Hebei (Bu and Gao 2017) and for the first time in Tibet in this paper.

#### Prabhergia imadatei

Taxon classificationAnimaliaCollembolaTullbergiidae

Tamura & Zhao, 1996

##### Material examined.

1 female (slide no. XZ-C2015071) (SNHM), China, Tibet, Motuo county, Dexing town, extracted from soil samples in broad-leaved forest, sample 4, Alt. 1480 m, 29°45'N, 95°28'E, 3-XI-2015, coll. Y. Bu & G. Yang. Holotype (SEM). female, China, Yunnan, Mengla county, Menglun town, Xishuangbanna Tropical Botanic Garden, Alt. 550 m, 28-X-1992. Paratypes (SEM), 4 females, same data as for holotype.

##### Distribution.

The species was only known from its type locality in Yunnan Province, southeast China. It is here recorded for the first time in Tibet.

##### Remarks.

The description of dorsal chaetotaxy of *Prabhergia imadatei* in the original paper contains some errors, such as nominations and numbers of anterior and medial setae on Th. II and III, Abd. I–V. The type specimens were examined and a revised dorsal chaetotaxy of the species is given below in Table 2.

**Table 2.** Dorsal Chaetotaxy of *Prabhergia imadatei* (revised).

Row	Thorax			Abdomen				
	I	II	III	I	II	III	IV	V
a	-	10	10	10	10	10	8^3^	10^5^
m	8	6^1^	6^1^	2^2^	2^2^	2^2^	2^4^	-
p	-	8	8	10	10	10	10	6^6^
pl	2	3	3	2	2	2	6	1

^1^ seta m1, m4, m5 present; ^2^ seta m3 present; ^3^ seta a5 absent; ^4^ seta m4 present; ^5^ seta a2 and a4 as macroseta; ^6^ setae p2 and p4 absent, sensory seta p3 slightly differentiated.

## Supplementary Material

XML Treatment for Metaphorura

XML Treatment for Metaphorura motuoensis

XML Treatment for Mesaphorura yosii

XML Treatment for Mesaphorura hylophila

XML Treatment for Prabhergia imadatei
